# Pathogenic copy number variations are associated with foetal short femur length in a tertiary referral centre study

**DOI:** 10.1111/jcmm.17821

**Published:** 2023-07-03

**Authors:** Meiying Cai, Yanting Que, Meihuan Chen, Min Zhang, Hailong Huang, Liangpu Xu, Na Lin

**Affiliations:** ^1^ Medical Genetic Diagnosis and Therapy Center, Fujian Maternity and Child Health Hospital College of Clinical Medicine for Obstetrics & Gynecology and Pediatrics, Fujian Medical University, Fujian Key Laboratory for Prenatal Diagnosis and Birth Defect Fuzhou China; ^2^ College of Clinical Medicine for Obstetrics and Gynecology and Pediatrics Fujian Medical University Fuzhou China

**Keywords:** 7q11.23 microdeletion, chromosomal microarray analysis, copy number variation, fetal short femur, genetic abnormality, ultrasonography

## Abstract

Shortened foetal femur length (FL) is a common abnormal phenotype that often causes anxiety in pregnant women, and standard clinical treatments remain unavailable. We investigated the clinical characteristics, genetic aetiology and obstetric pregnancy outcomes of foetuses with short FL and provided a reference for perinatal management of such cases. Chromosomal microarray analysis was used to analyse the copy number variations (CNV) in short FL foetuses. Of the 218 foetuses with short FL, 33 foetuses exhibited abnormal CNVs, including 19 with pathogenic CNVs and 14 with variations of uncertain clinical significance. Of the 19 foetuses with pathogenic CNVs, four had aneuploidy, 14 had deletions/duplications, and one had pathogenic uniparental diploidy. The 7q11.23 microdeletion was detected in three foetuses. The severity of short FL was not associated with the rate of pathogenic CNVs. The duration of short FL for the intrauterine ultrasound phenotype in foetuses carrying a pathogenic CNV was independent of the gestational age. Further, maternal age was not associated with the incidence of foetal pathogenic CNVs. Adverse pregnancy outcomes occurred in 77 cases, including termination of pregnancy in 63 cases, postnatal dwarfed foetuses with intellectual disability in 11 cases, and three deaths within 3 months of birth. Pathogenic CNVs closely related to foetal short FL were identified, among which the 7q11.23 microdeletion was highly associated with short FL development. This study provides a reference for the perinatal management of foetuses with short FL.

## 1 INTRODUCTION

Foetal femur length (FL) is a primary ultrasound measure used to assess foetal growth and skeletal development during pregnancy. Short FL is a common abnormal phenotype in foetuses, which often causes anxiety in pregnant women, and ultrasonographic diagnostic criteria for short FL have not yet been standardized. Short FL has been defined based on several criteria, including foetal FL below the 5th percentile^1^, ^2^ or below two standard deviations (SDs),^3^ a difference between foetal biparietal diameter and FL >4 cm (measured via ultrasound),^4^ or a FL/foot length <0.88.^5^ Foetal FL varies considerably depending on race, ethnicity, nutritional status and congenital diseases. Common causes of short FL include physiological short FL, foetal growth restriction (FGR), congenital skeletal hypoplasia and genomic abnormalities,^6^, ^7^ and obstetricians often face challenges in providing clinical treatment. Thus, it is necessary to establish diagnostic indicators, conduct timely prenatal diagnoses, identify various possible pathological factors that shorten FL and conduct foetus risk assessments to provide appropriate treatment.

Conventional karyotyping has low sensitivity and is a time‐consuming method for detecting inherited disorders. Chromosomal microarray analysis (CMA) is an effective method for detecting chromosomal microdeletions and microduplications at the genome level. Owing to its rapid, high‐throughput and high‐resolution capability, it is considered a powerful tool for the diagnosis of developmental and intellectual disabilities, autism and multiple congenital anomalies^8^, ^9^, ^10^; however, its efficiency in the prenatal diagnosis of short FL remains to be investigated. Therefore, we performed CMA to analyze the copy number variations (CNVs) in 218 foetuses with short FL and investigated their clinical characteristics, genetic aetiology and obstetric pregnancy outcomes to provide a reference for perinatal management of such cases.

## 2 MATERIALS AND METHODS

### 2.1 Patient data

This study enrolled 218 pregnant women who received a prenatal diagnosis of short FL via foetal ultrasound at a tertiary care centre at the Medical Genetic Diagnosis and Therapy Center of Fujian Maternal and Child Health Hospital between January 2016 and August 2021. The gestational age range was 18–37 weeks, and the maternal age range was 17–49 years. Depending on the gestational age, amniotic fluid or cord blood samples were collected for CMA; amniotic fluid was collected via amniocentesis at a gestational age of 18–24 weeks, whereas umbilical cord blood was collected via cordocentesis at a gestational age > 24 weeks. The inclusion criteria were as follows: gestational age determined based on the date of last menstruation, detailed menstrual history and ultrasound examination during the first trimester. Gestational age was determined based on the guidelines set forth by the American Congress of Obstetricians and Gynaecologists,^11^ the Society of Obstetricians and Gynaecologists of Canada,^12^ and expert consensus. Short FL was defined as an ultrasound measurement below 2SDs of the mean FL of the given gestational age. This study was approved by the Ethics Committee of Fujian Maternity and Child Health Hospital. Written informed consent was obtained from all participants.

### 2.2 Chromosomal microarray analysis

For amniotic fluid collection, approximately 20 mL of amniotic fluid was collected using ultrasound‐guided amniocentesis, whereas for umbilical cord blood collection, 1–2 mL of umbilical cord blood were collected using ultrasound‐guided cordocentesis. Genomic DNA was extracted from the amniotic fluid or foetal umbilical cord blood cells using the QIAamp DNA Blood Mini Kit (Qiagen) and then digested, amplified, purified, fragmented, labelled and hybridized to the Affymetrix CytoScan 750KSNP Array (Affymetrix). All data analyses were performed using Chromosome Analysis Suite version 3.2. The sequence reference version was CRCh37/hg19. According to the probe design of the microarray, an average of one probe per 4 kb is covered, with encrypted coverage of major genes. For accurate results, all CNVs were analysed at the resolution of 100 kb of 50 markers. The CNVs were evaluated using public databases, including the database of genomic variants (http://dgv.tcag.ca/dgv/app/home), DECIPHER database (https://decipher.sanger.ac.uk/), Online Mendelian Inheritance in Man (https://www.omim.org/), International Standards for Cytogenomic Arrays Consortium (http://www.iscaconsortium.org/), Cytogenomics Array Group CNV Database (http://www.cagdb.org/) and National Center for Biotechnology Information (National Institutes of Health, Bethesda). All detected CNVs were classified as pathogenic, likely pathogenic, variants of uncertain clinical significance (VUS), likely benign CNVs or benign CNVs according to the American College of Medical Genetics standards and guidelines.^13^ Uniparental disomy (UPD) is defined as a chromosomal region/segment from one parent being replaced by a homologous part from the other parent, or an individual having both homologous chromosomes from the same parent. Peripheral blood was also sampled from the parents of foetuses with VUS for CMA analysis to verify whether the variant represented a de novo CNV or whether it was inherited from the father or mother.

### 2.3 Follow‐up and pregnancy outcomes

All patients were followed up by telephone regarding the pregnancy outcomes and postnatal conditions. The follow‐ups obtained data on postnatal physical growth and neurobehavioural development. All adverse pregnancy outcomes, such as miscarriage, stillbirths, intrauterine growth retardation, developmental abnormalities and perinatal or infant death, were recorded.

### 2.4 Statistical analysis

IBM SPSS Statistics for Windows version 25 (IBM) was used for all statistical analyses. Chi‐squared tests or Fisher's exact tests were used to compare categorical variables between groups. Statistical significance was set at *p* < 0.05.

## 3 RESULTS

### 3.1 Clinical characteristics of enrolled cases

A total of 218 cases were included in this study, including 52 isolated FL foetuses, 45 FL foetuses with abnormal ultrasound structure and 121 FL foetuses with abnormal ultrasound soft indicators. Karyotype analysis and CMA were performed in 218 samples, including 80 samples of amniotic fluid puncture and 138 samples of umbilical cord blood puncture. The mean age of pregnant women was 28 ± 4 years, and the mean gestational age was 24 ± 5 weeks.

### 3.2 Chromosomal microarray analysis

CMA was performed for 218 samples with a success rate of 100%. Of the 218 foetuses with short FL, 33 foetuses were detected to have abnormal CNVs (15.1%, 33/218), including 19 foetuses with pathogenic CNVs (8.7%, 19/218; Table 1) and 14 with VUS (6.4%, 14/218; Table 2). Of the 19 foetuses with pathogenic CNVs, four had aneuploidy (two cases with trisomy 21 syndrome; one case with trisomy 18 syndrome; and one case with XXX), 14 had deletions/duplications (three cases with 7q11.23 microdeletions, three cases with 4p16.3 microdeletions, three cases with Xp22.33 microdeletions, and one case each with Turner syndrome, 9q33.3 microdeletion, 5p15.33p11 duplication, 7q31.31q36.3 duplication and 9p24.3q13 duplication) and one had pathogenic uniparental diploidy (UPD) at 6p25.3q27.

**TABLE 1:** Pathogenic copy number variations (CNVs) in foetuses with short femur length (FL).

Case	Ultrasound phenotype	CMA result	Size (Mb)	Dosage sensitivity syndrome/gene	Inheritance	Obstetrical outcome
1	FL below 2–4 SDs	Trisomy 21	–	Trisomy 21 syndrome	–	TP
2	FL below 2–4 SDs, FGR, congenital heart disease, nasal dysplasia, parenchymal echogenicity in both kidneys, intestinal echogenicity	Trisomy 21	–	Trisomy 21 syndrome	–	TP
3	FL below 2–4 SDs, FGR, ventricular septal defect, large pulmonary artery to aorta ratio, nasal dysplasia, overlapping fingers	Trisomy 18		Trisomy 18 syndrome	–	TP
4	FL below 2–4 SDs, FGR	Trisomy X	–	Trisomy X syndrome	–	TP
5	FL below 2–4 SDs	7q11.23microdeletion	1.5	Williams–Beuren syndrome	–	TP
6	FL below 2–4 SDs	7q11.23microdeletion	1.5	Williams–Beuren syndrome	–	TP
7	FL below 2–4 SDs, FGR	7q11.23microdeletion	1.4	Williams–Beuren syndrome	De novo	TP
8	FL below 2–4 SDs, FGR, smaller kidneys, nasal dysplasia	4p16.3p15.1deletion	35	Wolf–Hirschhorn syndrome	–	TP
9	FL below 2–4 SDs, FGR, umbilical artery stenosis	4p16.3p16.1microdeletion	6.5	Wolf–Hirschhorn syndrome	–	TP
10	FL below 2–4 SDs, FGR, nasal dysplasia, ventricular septal defect, persistent superior vena cava, small kidneys	4p16.3microdeletion	3.2	Wolf–Hirschhorn syndrome	–	TP
11	FL below 2–4 SDs	Xp22.33microdeletion	2.8	Leri‐Weill dyschondrosteosis	–	TP
12	FL below 2–4 SDs	Xp22.33microdeletion	0.5	Leri‐Weill dyschondrosteosis	–	TP
13	FL below 4 SDs, HL below 3 SD	Xp22.33microdeletion	**0.7**	Leri‐Weill dyschondrosteosis	–	TP
14	FL below 2–4 SDs, right dominant heart, right ventricular hypertrophy, mild tricuspid valve, pericardial effusion	PartialXp11.3q13.3deletion	42.5	Partial Turner syndrome	–	TP
15	FL below 4SDs, nasal dysplasia, HL below 4SDs	9q33.3microdeletion	2.8	LMX1B	–	TP
16	FL below 2–4 SDs, brain development inconsistent with GA	5p15.33p11 duplication	45.8	Partial trisomy 5 syndrome	–	TP
17	FL below 2–4 SDs, midline brain structure	7q31.31q36.3duplication	40	7q36.3 ZRS (SHH cis‐regulatory) duplication region (within LMBR1 intron 5), Xq28 recurrent region (int22h1/int22h2‐flanked) (includes RAB39B)	–	TP
18	FL below 2–4 SDs, FGR	9p24.3q13 duplication	68	Partial tetrasomy 9 syndrome	–	TP
19	FL below 4SDs, FGR, mild tricuspid valve, inverted alpha wave of venous catheter	6p25.3q27 UPD	–	Transient neonatal diabetes mellitus	Paternal	TP

Abbreviations: CMA, chromosomal microarray analysis; SDs, standard deviations; FGR, fetal growth restriction; TP, termination of pregnancy; UPD, uniparental diploidy.

**TABLE 2:** Variants of uncertain clinical significance (VUS) copy number variations (CNVs) in foetuses with short femur length (FL).

Case	Ultrasound phenotype	CMA result	Size (Mb)	Dosage sensitivity syndrome/gene	Inheritance	Obstetrical outcome
1	FL below 2–4 SDs	7p21.2 microdeletion	2.4	–	De novo	Healthy
2	FL below 2–4 SDs	17q21.31 microduplication	0.7	–	–	No walking
3	FL below 2–4 SDs	22q12.3q13.33homozygous	16	–	–	Healthy
4	FL below 2–4 SDs	6q12q14.1 homozygous	12.9	–	–	TP
5	FL below 2–4 SDs, HC below3.1 SDs	19q13.42q13.43microduplication	1.3	–	De novo	Healthy
6	FL below 2–4 SDs, FGR	17q24.3 microduplication	1.2	–	Maternal	Healthy
7	FL below 2–4 SDs, FGR	18p11.23p11.22 microduplication	1.8	–	Paternal	Healthy
8	FL below 2–4 SDs, FGR, persistent left superior vena cava, narrow inner diameter of aortic arch	2p25.3p11.2homozygous	–	–	Maternal	Healthy
9	FL below 2–4 SDs, HC below 2 SDs	10q11.22q11.23 microduplication	5.6	*–*	De novo	Healthy
10	FL below 2–4 SDs, absent inferior cerebellar vermis, HL below 2SD	22q11.21 microdeletion	0.4	22q11.2 recurrent region (central, B/C‐D)/CRKL	*–*	TP
11	FL below 2–4 SDs	16q24.3 microdeletion	0.2	*–*	*–*	TP
12	FL below 2–4 SDs, slightly widened left lateral ventricle in fetus, HL below 2SD	2q13 microdeletion	1.8	2q13 recurrent region (includes BCL2L11)	–	TP
13	FL below 2–4 SDs, FGR, single umbilical artery, oligohydramnios, placental thickening	8q11.23q12.1 microdeletion	5.1	*–*	*–*	TP
14	FL below 2–4 SDs, FGR, ventricular septal defect, aortic stenosis	16q23.2q24.3homozygous, 16p13.3p12.3 homozygous	10.3	*–*	Maternal	TP

Abbreviations: CMA, chromosomal microarray analysis; SDs, standard deviations; FGR, fetal growth restriction, HL: humerus length; HC, head circumference.

### 3.3 Subgroup analysis according to the presence of other ultrasonographic abnormalities

Foetuses with short FL were classified into three groups according to the presence of other ultrasonographic abnormalities: isolated short FL, short FL with ultrasonographic soft markers and short FL with ultrasonographic structural anomalies. The rates of pathogenic CNVs in foetuses with isolated short FL, short FL with ultrasonographic structural malformations and short FL with ultrasonographic soft markers were 9.6% (5/52), 11.1% (5/45) and 7.4% (9/121), respectively. No significant differences were observed in the rate of pathogenic CNVs among the three groups **(**χ^2^ = 0.626, *p* = 0.731; Table 3).

**TABLE 3:** Subgroup analysis of 218 foetuses with short femur length (FL).

Group	Total number of cases	Pathogenic CNV (number of cases)	Pathogenic CNV rate (%)
Ultrasound phenotyping			
Isolated short FL	52	5	9.6
Short FL with ultrasound structural abnormalities	45	5	11.1
Short FL with ultrasonographic soft markers	121	9	7.4
Degree of femoral shortening			
−2SDs < FL ≤ −4SDs	191	16	8.4
FL < −4SDs	27	3	11.1
Short FL at initial diagnosis			
Second trimester	106	8	7.5
Third pregnancy	112	11	9.8
Maternal age group			
Younger age group	196	15	7.7
Advanced age group	22	4	18.2

Abbreviations: CNV, copy number variation; SDs, standard deviations.

### 3.4 Subgroup analysis with respect to the degree of femoral shortening

Foetuses with short FL were classified into two groups according to the degree of femoral shortening: those with relatively mild shortening (‐2SD < FL ≤ −4SD) and those with severe shortening (FL < −4SD). The rates of pathogenic CNVs in the mild and severe FL shortening groups were 8.4% (16/191) and 11.1% (3/27), respectively. No significant difference was observed in the rate of pathogenic CNVs between the two groups **(**χ^2^ = 0.222, *p* = 0.895; Table 3).

### 3.5 Subgroup analysis of different gestational ages at the initial diagnosis of short FL

Foetuses with short FL were classified into two groups according to their gestational age at the time of initial diagnosis: those presenting with short FL in the second trimester and those presenting with short FL in the third trimester. In total, 106 foetuses were initially diagnosed with short femurs in the second trimester (gestational age range, 16–28 weeks), and the remaining 112 foetuses were diagnosed in the third trimester (gestational age, ≥28 weeks). The rate of pathogenic CNVs was 7.5% (8/106) for foetuses diagnosed in the second trimester and 9.8% (11/112) for those diagnosed in the third trimester. No significant difference was observed in the rate of pathogenic CNVs between the two groups (χ^2^ = 0.354, *p* = 0.552; Table 3).

### 3.6 Subgroup analysis with respect to maternal age

Foetuses with short FL were classified into two groups according to maternal age: those <35 and those ≥35 years of age. The rate of pathogenic CNVs in the foetuses of younger mothers (<35 years old) was 7.7% (15/196), whereas that of the older mothers was 18.2% (4/22). No significant difference was observed in the rate of pathogenic CNVs between the two groups (*p* = 0.109; Table 3).

### 3.7 Follow‐ups and obstetrical outcomes

Attempts were made to follow‐up with all 218 cases to assess the outcomes and document postnatal development, with successful follow‐up with 199 cases, whereas the remaining 19 cases were lost to follow‐up. All the 19 cases lost to follow‐up had negative CMA results. Of the 166 cases with negative CMA results successfully followed up, 38 were terminated, 115 were healthy after birth, three died after birth and 10 had developmental delays after birth. Of the 199 follow‐ups, the overall rate of adverse pregnancy outcomes was 38.7% (77/199). Of the 77 cases with adverse pregnancy outcomes, 63 pregnancies were terminated, 11 were dwarfs accompanied by intellectual disability after birth and three infants died within 3 months of birth. Of the 63 cases that were terminated, 38 foetuses had normal microarray results (three cases with isolated FL and 35 cases with non‐isolated FL), 19 had pathogenic CNVs and six had VUS. The remaining 122 foetuses exhibited normal growth and development after birth (115 cases with normal CMAs and seven with VUS; Table 4).

**TABLE 4:** Pregnancy outcomes of 218 foetuses with short femur length (FL).

CMA	Healthy	TP	Pregnancy postnatal death	Outcome developmental delay	Lost to follow‐up	Total
Normal	115	38	3	10	19	185
pCNV	0	19	0	0	0	19
VUS	7	6	0	1	0	14

Abbreviations: CMA, chromosomal microarray analysis; CNV, copy number variation; pCNV, pathogenic CNV; TP, termination of pregnancy; VUS, variants of uncertain clinical significance.

## 4 DISCUSSION

Foetal FL is routinely measured in pre‐ultrasound examinations to estimate the gestational age and assess intrauterine growth and development. Short FL may be caused by congenital skeletal malformation, chromosomal abnormalities, FGR and other pathological factors, as well as by genetic factors or calculation errors, in normal infants with low feeding behaviour, etc. Thus, it is difficult to perform a differential diagnosis based on ultrasonography alone, and an invasive prenatal diagnosis is required to identify the aetiology. In the present study, 218 foetuses with short FL diagnosed via ultrasonography were analysed for CNVs using CMA to investigate the clinical features, genetic aetiology and obstetric pregnancy outcomes and provide a reference for the perinatal management of such cases.

CMA, also known as molecular karyotyping, enables genome‐wide scanning and detection of unbalanced rearrangements, such as small deletions and duplications in the genome, most UPDs and triploidies and chimerism, to a certain extent.^14^, ^15^ Indeed, we detected 19 cases of pathogenic CNVs, with a detection rate of 8.7%. Previous studie have reported chromosomal abnormalities in 6%–8% of foetuses with a short FL,^16^, ^17^, ^18^ which was consistent with our findings. Femoral shortness is a soft ultrasound indicator of foetal chromosomal aneuploidy, and the sensitivity of short FL screening for trisomy 21 is 24%, with a false positivity rate of 4.7%.^19^, ^20^ In the present study, of the 19 cases with pathogenic CNVs, four had aneuploidy (two cases of trisomy 21; one case of trisomy 18; and one case of XXX). Furthermore, three cases of Xp22.33 microdeletion were detected in this study. Microdeletions in the Xp22.33 region disrupt the short‐stature homeobox (*SHOX*) gene,^21^, ^22^ which can result in a short stature or Leri–Weill chondro‐osteogenesis disorder (characterized by varying degrees of short stature, short forearm, arcuate radius, disorganized carpal bones, Madelung deformity, limited joint movement, short leg and other abnormalities).^23^, ^24^ Additionally, the 4p16.3 deletion was detected in three foetuses, all of whom presented with FGR in addition to short FL. Microdeletion in the 4p16.3 region can lead to the development of the Wolf–Hirschhorn syndrome,^25^ which is characterized by prenatal FGR, postnatal FGR, intellectual disabilities, characteristic facial deformities (such as armour‐like facial features) and congenital abnormalities (such as heart and kidney malformations).^26^ In the present study, the 7q11.23 microdeletion was detected in three foetuses. This microdeletion can lead to the development of the Williams–Beuren syndrome (WBS).^27^, ^28^ The main clinical phenotypes of patients with WBS include cardiovascular malformations, intellectual disabilities, craniofacial abnormalities, connective tissue abnormalities, low body weight, hypotonia and other congenital abnormalities.^29^ Previous studies on WBS have not mentioned the presence of FGR or a clinical growth retardation phenotype in the patients; however, the ultrasound phenotypes of all three foetuses in the present study revealed a short FL, indicating that the gene in the 7q11.23 microdeletion region is highly correlated with the development of FL and demonstrating that our study provides additional phenotypes for updating the current databases. Additionally, single cases of 9q33.3 microdeletion, 5p15.33p11 duplication, 7q31.31q36.3 duplication and 9p24.3q13 duplication were detected in the present study, all of which have been reported to be associated with growth retardation. Moreover, one fetus in the present study had UPD. A previous study reported an association between paternal 6p25.3q27 UPD and foetal growth retardation.^30^ Altogether, our study provides insights into the pathogenic CNVs underlying the phenotypes associated with short foetal FL.

Parilla et al.^31^ proposed that premature and severe short limb malformations are indicative of fatal skeletal dysplasia. Based on the degree of FL shortening and diagnosis, short FL with fatal skeletal abnormalities is associated with an early occurrence time and tends to be severe. However, in the present study, neither the gestational age at the time of initial diagnosis nor the severity of short FL were associated with the rate of pathogenic CNVs. Further, maternal age was not associated with the incidence of foetal pathogenic CNVs. Conversely, fetal FL shortening was closely associated with chromosomal abnormalities.

Our results showed that the overall rate of adverse pregnancy outcomes among the cases that were successfully followed up was 38.7% (77/199). The main reason for the termination of pregnancy was short FL combined with other ultrasound abnormalities and was related to pathogenic CNVs. Interestingly, 14 adverse pregnancy outcomes lacking pathogenic CNVs were observed, suggesting the presence of other abnormalities, such as those associated with genetic mutations. Similarly, foetuses with short femurs and negative CMA results may have had certain genetic mutations. Hence, whole exon sequencing should be carried out in foetuses with short femur and negative CMA results (especially those with extremely short femur). Notably, 122 foetuses were generally normal after birth, indicating that normal pregnancy outcomes are not uncommon in foetuses with a short FL when CMA indicates the presence of VUS. In the present study, seven foetuses with VUS, including 7p21.2 microdeletion, 19q13.42q13.43 microduplication, 17q24.3 microduplication, 18p11.23p11.22 microduplication, 10q11.22q11.23 microduplication, 2p25.3p11.2 homozygous and 22q12.3q13.33 homozygous, were generally normal after birth and their parents were healthy. At present, there is no relevant report on these seven cases of VUS in the literature and databases. Although the current growth and development assessments of these 122 foetuses were normal, long‐term follow‐ups will be informative for genetic counselling in the future.

This study was limited by a small sample size, with only 218 foetuses with short FL retrospectively analysed, as well as our reliance on CMA because of the lack of suitable methods for data analysis. Furthermore, while the chromosomal pathogenic causes of short FL were excluded, genetic mutations were not and may have influenced the results. Indeed, it has been reported that short FL may be associated with congenital skeletal malformations, among which mutations in fibroblast growth factor receptor 3 are the most common.^17^, ^32^ Therefore, future studies should evaluate larger cohorts and perform single‐gene detection to improve the perinatal management of foetuses with short FL.

In conclusion, this study analysed the aetiology of CNVs in foetuses with short FL and identified pathogenic CNVs that were closely related to short FL, including a microdeletion in 7q11.23. Further, this study analysed the clinical characteristics and follow‐up of foetuses with short FL to provide a reference for perinatal management of such foetuses. Our findings provide valuable information for the evaluation and management of foetuses with short femurs by obstetricians, allowing them to provide good consultation to prospective parents.

## AUTHOR CONTRIBUTIONS

**Meiying Cai:** Writing – original draft (equal). **yanting que:** Data curation (equal); formal analysis (equal). **Meihuan Chen:** Validation (equal). **min zhang:** Methodology (equal). **Hailong Huang:** Supervision (equal). **liangpu xu:** Visualization (equal). **Na Lin:** Investigation (equal); visualization (equal).

## CONFLICT OF INTEREST STATEMENT

The authors declare no conflict of interest.

## Data Availability

Data supporting the results of this study can be obtained from the corresponding author on request.

## References

[1] Vermeer N , Bekker MN . Association of isolated short fetal femur with intrauterine growth restriction. Prenat Diagn. 2013;33:365‐370. 23440724 10.1002/pd.4068

[2] Mathiesen JM , Aksglaede L , Skibsted L , Petersen OB , Tabor A . Outcome of fetuses with short femur length detected at second‐trimester anomaly scan: a national survey. Ultrasound Obstet Gynecol. 2014;44:160‐165. 24357398 10.1002/uog.13286

[3] Morales‐Roselló J , Llorens NP . Outcome of fetuses with diagnosis of isolated short femur in the second half of pregnancy. ISRN Obstet Gynecol. 2012;2012:1‐5. 10.5402/2012/268218 PMC3345228 22577572

[4] Papageorghiou AT , Fratelli N , Leslie K , Bhide A , Thilaganathan B . Outcome of fetuses with antenatally diagnosed short femur. Ultrasound Obstet Gynecol. 2010;31:507‐511. 10.1002/uog.5265 18286672

[5] Harper LM , Gray D , Dicke J , Stamilio DM , Odibo AO . Do race‐specific definitions of short long bones improve the detection of down syndrome on second‐trimester genetic sonograms? J Ultrasound Med. 2010;29:231‐235. 20103793 10.7863/jum.2010.29.2.231

[6] Zhang Y , Meng H , Jiang Y , et al. Chinese fetal biometry: reference equations and comparison with charts from other populations. J Matern Fetal Neonatal Med. 2019;32(9):1507‐1515. 29216774 10.1080/14767058.2017.1410787

[7] Kasraeian M , Shahraki HR , Asadi N , Vafaei H , Sameni S . Cross‐sectional study of fetal long‐bone length in an Iranian population at 17–25weeks of gestation. Int J Gynecol Obstet. 2017;137:20‐25. 10.1002/ijgo.12099 28083947

[8] Cappuccio G , Vitiello F , Casertano A , et al. New insights in the interpretation of array‐CGH: autism spectrum disorder and positive family history for intellectual disability predict the detection of pathogenic variants. Ital J Pediatr. 2016;42:39‐45. 27072107 10.1186/s13052-016-0246-7 PMC4830019

[9] Miller DT , Adam MP , Aradhya S , et al. Consensus statement: chromosomal microarray is a first‐tier clinical diagnostic test for individuals with developmental disabilities or congenital anomalies. Am J Hum Genet. 2010;86(5):749‐764. 20466091 10.1016/j.ajhg.2010.04.006 PMC2869000

[10] Syrmou A , Tzetis M , Fryssira H , et al. Array comparative genomic hybridization as a clinical diagnostic tool in syndromic and nonsyndromic congenital heart disease. Pediatr Res. 2013;73(6):772‐776. 23481551 10.1038/pr.2013.41

[11] Committee opinion, number 611. Method for estimating due date. Obstet Gynecol. 2014;124(4):863‐866. 25244460 10.1097/01.AOG.0000454932.15177.be

[12] Butt K , Lim K , Bly S , et al. Determination of gestational age by ultrasound. J Obstet Gynaecol Can. 2014;36(2):171‐181. 24518917 10.1016/S1701-2163(15)30664-2

[13] Riggs ER , Andersen EF , Cherry AM , et al. Technical standards for the interpretation and reporting of constitutional copy‐number variants: a joint consensus recommendation of the American College of Medical Genetics and Genomics (ACMG) and the Clinical Genome Resource (ClinGen). Genet Med. 2020;22(2):245‐257. 31690835 10.1038/s41436-019-0686-8 PMC7313390

[14] Brady PD , Vermeesch JRJP . Genomic microarrays: a technology overview. Prenat Diagn. 2012;32(4):336‐343. 22467164 10.1002/pd.2933

[15] Zhang Y , Zhong M , DJJoC Z , Medicine M . Chromosomal mosaicism detected by karyotyping and chromosomal microarray analysis in prenatal diagnosis. J Cell Mol Med. 2021;25(1):358‐366. 33201576 10.1111/jcmm.16080 PMC7810963

[16] Ren Y , You YQ , Zhou HH , Wang LX , Lu YP . Clinical analysis of 21 cases with short fetal femur in the third trimester. Zhonghua Fu Chan Ke Za Zhi. 2017;52(2):86‐92. 28253570 10.3760/cma.j.issn.0529-567X.2017.02.004

[17] Li Q , Zhang Z , Wang J , et al. Prenatal diagnosis of genetic aberrations in fetuses with short femur detected by ultrasound: a prospective cohort study. Prenat Diagn. 2021;41(9):1153‐1163. 34185917 10.1002/pd.6006

[18] Liu J , Huang L , He Z , Lin S , Luo Y . Clinical value of genetic analysis in prenatal diagnosis of short femur. Mol Genet Genomic Med. 2019;7(11):e97. 10.1002/mgg3.978 PMC6825856 31566912

[19] Drugan A , Johnson MP , Evans MI . Ultrasound screening for fetal chromosome anomalies. Am J Med Genet. 2000;90(2):98‐107. 10607945 10.1002/(sici)1096-8628(20000117)90:2<98::aid-ajmg2>3.0.co;2-h

[20] Nyberg DA , Resta RG , Luthy DA , Hickok DE , Williams MA . Humerus and femur length shortening in the detection of Down's syndrome. Am J Obstet Gynecol. 1993;168(2):534‐538. 8438923 10.1016/0002-9378(93)90487-4

[21] Cho SY , Ki CS , Jang JH , et al. Familial Xp22.33‐Xp22.12 deletion delineated by chromosomal microarray analysis causes proportionate short stature. Am J Med Genet A. 2012;158A(6):1462‐1468. 22581654 10.1002/ajmg.a.35357

[22] Fabiola D , Chan JT , Hunain A , Roxana AC , Lenika DS , Zohra S . Turner syndrome due to Xp22.33 deletion with preserved gonadal function: case report. Oxf Med Case Reports. 2019;2019(5):omz028. 31214355 10.1093/omcr/omz028 PMC6570789

[23] Sadler B , Haller G , Antunes L , Nikolov M , Gurnett CA . Rare and de novo duplications containing SHOX in clubfoot. J Med Genet. 2020;57(12):851‐857. 32518174 10.1136/jmedgenet-2020-106842 PMC7688552

[24] Fukami M , Seki A , Ogata T . SHOX Haploinsufficiency as a cause of syndromic and nonsyndromic short stature. Mol Syndromol. 2016;7(1):3‐11. 27194967 10.1159/000444596 PMC4862394

[25] Zheng W , Chen B , Yin Z , Huang X , Liang Y . Identification of a critical region on chromosome 4p16.3 for Wolf‐Hirschhorn syndrome‐associated fetal growth retardation. Zhonghua Yi Xue Yi Chuan Xue Za Zhi. 2020;37(7):731‐735. 32619252 10.3760/cma.j.issn.1003-9406.2020.07.007

[26] Bi W , Cheung SW , Breman AM , Bacino CA . 4p16.3 microdeletions and microduplications detected by chromosomal microarray analysis: new insights into mechanisms and critical regions. Am J Med Genet A. 2016;170(10):2540‐2550. 27287194 10.1002/ajmg.a.37796

[27] Mulle JG , Pulver AE , McGrath JA , et al. Reciprocal duplication of the Williams‐Beuren syndrome deletion on chromosome 7q11.23 is associated with schizophrenia. Biol Psychiatry. 2014;75(5):371‐377. 23871472 10.1016/j.biopsych.2013.05.040 PMC3838485

[28] Alesi V , Loddo S , Orlando V , et al. Atypical 7q11.23 deletions excluding ELN gene result in Williams–Beuren syndrome craniofacial features and neurocognitive profile. Am J Med Genet A. 2021;185(1):242‐249. 33098373 10.1002/ajmg.a.61937

[29] Yau EK , Lo IF , Lam ST . Williams‐Beuren syndrome in the Hong Kong Chinese population: retrospective study. Hong Kong Med J. 2004;10(1):22‐27. 14967851

[30] Lazier J , Martin N , Stavropoulos JD , Chitayat D . Maternal uniparental disomy for chromosome 6 in a patient with IUGR, ambiguous genitalia, and persistent mullerian structures. Am J Med Genet A. 2016;170(12):3227‐3230. 27500688 10.1002/ajmg.a.37876

[31] Parilla BV , Leeth EA , Kambich MP , Chillis P , MacGregor SM . The antenatal detection of skeletal dysplasias. J Ultrasound Med. 2003;22(3):255‐258. 12636325 10.7863/jum.2003.22.3.255

[32] Bellus GA . Achondroplasia is defined by recurrent G380R mutations of FGFR3. Am J Hum Genet. 1995;56(2):368‐373. 7847369 PMC1801129

